# Editorial: Microbial associates of blood-sucking arthropods and other animals: relevance to their physiology, ecology and evolution

**DOI:** 10.3389/fmicb.2023.1256275

**Published:** 2023-07-26

**Authors:** Takema Fukatsu, Yuval Gottlieb, Olivier Duron, Joerg Graf

**Affiliations:** ^1^Bioproduction Research Institute, National Institute of Advanced Industrial Science and Technology, Tsukuba, Japan; ^2^Department of Biological Sciences, Graduate School of Science, The University of Tokyo, Tokyo, Japan; ^3^Graduate School of Life and Environmental Sciences, University of Tsukuba, Tsukuba, Japan; ^4^The Robert H. Smith Faculty of Agriculture, Food and Environment, Koret School of Veterinary Medicine, The Hebrew University of Jerusalem, Rehovot, Israel; ^5^MIVEGEC, CNRS, IRD, University of Montpellier, Montpellier, France; ^6^Department of Molecular and Cell Biology, University of Connecticut, Storrs, CT, United States; ^7^Pacific Biosciences Research Center, University of Hawai'i at Mānoa, Honolulu, HI, United States

**Keywords:** blood-feeding, insect, tick, mite, crustacean, leech, microbiome, B vitamin

Lice, bed bugs, ticks, leeches, and other tiny blood-sucking crawling creatures are regarded as nasty vampires, causing itches, eliciting disgusting feeling, vectoring human and animal diseases, and thereby bringing about medical, health, hygienic and mental problems in human societies (Lehane, 2005). Besides the microbial pathogens they carry and transmit, unique microorganisms are associated with them and affect their physiology, ecology, and other biological aspects in a variety of ways (Rio et al., 2016; Husnik, 2018). For example, their food, vertebrate blood, is certainly nutrition-rich, but devoid of some important nutrients like B vitamins. Hence, many blood feeders possess specialized organs called bacteriomes for hosting vitamin-provisioning symbionts (Buchner, 1965), which enable them to thrive only on the blood meal (Duron and Gottlieb, 2020). Fully engorged blood feeders exhibit a challenging gut environment with plenty of proteins, iron, heme and antimicrobial components such as antibodies and complements, which may foster unique gut microbiome (Sterkel et al., 2017).

Owing to recent development of high-throughput DNA sequencing technologies, our knowledge of the microbiomes associated with these blood-sucking invertebrates, which must be connected to their unique feeding habit and physiology, has been growing rapidly. Hence, this Research Topic “*Microbial Associates of Blood-Sucking Arthropods and Other Animals: Relevance to Their Physiology, Ecology and Evolution*” is aimed to provide a forum for new findings emerging in this research field. In total, nine articles and two reviews are compiled, which showcase the microbial associates of a diverse array of blood-feeding invertebrates including lice (Insecta: Psocodea), tsetse flies (Insecta: Diptera), fleas (Insecta: Siphonaptera), ticks (Arachnida: Ixodida) and mites (Arachnida: Mesostigmata) from the terrestrial ecosystem, and *Elthusa* and *Nerocila* (Crustacea: Isopoda), *Lernanthropus* (Crustacea: Copepoda) and fish leeches (Hirudinea: Piscicolidae) from the marine ecosystem.

Sucking lice (Psocodea: Anoplura) live on vertebrate blood as the sole food source throughout their life cycle (Durden and Musser, 1994), many of which possess specialized symbiotic organs for harboring specific symbiotic bacteria (Ries, 1931; Buchner, 1965). Both histological inspection and molecular phylogenetic survey revealed that their symbiotic organs and associated bacterial symbionts are strikingly diverse among different lice lineages and likely of independent evolutionary origins (Hypša and KriŽek, 2007; Boyd and Reed, 2012). In this Research Topic, three articles dealt with louse-associated symbiotic bacteria. Nishide, Oguchi, et al., investigated the endosymbiotic microbiota of the boar louse *Haematopinus apri*, identified a primary endosymbiont clade associated with the boar, swine and cattle lice, and designated it as “*Candidatus* Haematopinicola symbiotica”. Říhová et al. screened and assembled the metagenomic data of the chipmunk lice *Neohaematopinus* spp. and identified a genome-reduced endosymbiont designated as “*Candidatus* Lightella neohaematopini”. Doña et al. surveyed the microbiota associated with the seal louse *Echinophthirius horridus*, which uncovered diverse bacterial associates but failed to identify principal symbiotic bacteria. These reports highlight the dynamic evolutionary trajectories of the louse-microbe endosymbiotic associations entailing multiple and independent gains and losses.

Tsetse flies (Diptera: Glossinidae) are obligatory blood feeders distributed in sub-Saharan Africa, where they vector devastating human and animal pathogens *Trypanosoma* spp. (Krafsur, 2009). Tsetse flies are associated with a vitamin-provisioning primary symbiont *Wigglesworthia glossinidia*, a commensal bacterial associate *Sodalis glossinidius*, and a facultative endosymbiont *Wolbachia pipientis* (Aksoy, 2000). In the Research Topic, Lee et al. reviewed the current understanding of tsetse-microbe molecular interactions, with particular focus on recently accumulating knowledge about possible involvement of DNA methylation and microRNAs.

Fleas (Siphonaptera) are obligatory blood feeders of mammals and birds as adults, and notorious for vectoring *Yersinia pestis* and other pathogens (Bitam et al., 2010). Probably because their larvae live on organic debris without blood feeding, no obligatory microbial symbionts have been known from fleas, whereas diverse facultative bacterial associates have been detected, as Dong et al. identified *Wolbachia, Rickettsia* and *Bartonella* as the major bacterial associates of the fleas *Oropsylla silantiewi* and *Callopsylla dolabris* from Himalayan marmots. In the flea *Synosternus cleopatrae* from desert rodents, it was reported that, interestingly, *Wolbachia* infection is fixed in females but lacking or partial in males (Flatau et al., 2018). In this Research Topic, Flatau et al. treated *S. cleopatrae* with tetracycline and compared the life history parameters of *Wolbachia*-infected fleas with those of *Wolbachia*-free fleas, but no significant differences were detected between them.

Ticks (Ixodida: Ixodea) are obligatory blood feeders of terrestrial vertebrates including mammals, birds, reptiles and amphibians (Anderson and Magnarelli, 2008). Conventionally, ticks have been regarded as vectors of *Rickettsia, Coxiella* and other pathogens causing human and animal diseases (de la Fuente et al., 2008), but now it is widely recognized that ticks commonly host non-pathogenic, either commensalistic or mutualistic, microbial associates allied to *Coxiella, Rickettsia, Francisella, Midichloria, Wolbachia* and others (Bonnet et al., 2017). In this Research Topic, Hussain et al. reviewed such tick-microbe symbiotic continuum spanning from pathogens through commensals to mutualists. Dong et al. detected *Anaplasma, Wolbachia* and *Ehrlichia* as the major bacterial associates of the tick *Haemaphysalis qinghaiensis* from Himalayan marmots. Militzer et al. analyzed the effects of artificial feeding and antibiotic treatment on microbiome composition and fecundity of the tick *Ixodes ricinus* associated with *Midichloria, Rickettsia* and *Spiroplasma*.

The poultry red mite *Dermanyssus gallinae* (Mesostigmata: Dermanyssidae) is a blood sucking avian ectoparasite that often causes significant economic damage on poultry production (Sparagano et al., 2014). A previous study identified *Bartonella, Cardinium, Wolbachia* and *Rickettsiella* in European populations of *D. gallinae* (Hubert et al., 2017). In this Research Topic, Price et al. detected *Rickettsiella* from all 63 samples of *D. gallinae* derived from 63 localities across 15 European countries, and determined the 1.9 Mbp *Rickettsiella* genome that retains the synthetic pathways for thiamine (= vitamin B1), riboflavin (= vitamin B2) and pyridoxine (= vitamin B6). By contrast, Nishide, Sugimoto, et al., reported that, from 144 samples of *D. gallinae* collected from 18 poultry farms in Japan, *Bartonella, Cardinium, Wolbachia* and *Tsukamurella* were detected as major bacterial components, but *Rickettsiella* was not detected at all. These reports uncovered strikingly different microbiota across European and Japanese populations of *D. gallinae*. At present, whether *Rickettsiella* is the vitamin-provisioning primary symbiont of *D. gallinae* or not is elusive and to be established in future studies.

Finally, as the highlight of this Research Topic, Goffredi et al. reported the microbiomes of marine obligatory blood feeders that have been little investigated previously: fish ectoparasitic isopods *Elthusa vulgaris* and *Nerocila californica* (Isopoda: Cymothoidae); a fish ectoparasitic copepod *Lernanthropus latis* (Copepoda: Lernanthropidae); and fish leeches *Pterobdella occidentalis, Ostreobdella californiana*, and *Branchellion lobata* (Hirudinea: Piscicolidae). Interestingly, all the marine blood suckers exhibited peculiar gut microbiomes characterized by relatively low diversity dominated by *Vibrio* species.

In conclusion, the Research Topic presents an impressive overview of the current research coverage on the diversity of microbial symbioses among blood-sucking arthropods and other invertebrates. These reports significantly broaden our knowledge as to what types of microbiomes are associated with the obligatory blood feeders, and highlight the untouched research fields represented by, for example, marine obligatory blood feeders and their biological and functional aspects, as promising targets for future studies. It has been widely accepted that symbiotic interactions with microorganisms are essential for the ecology of insects, ticks, leeches and other invertebrates with obligatory blood feeding habit. Diverse bacterial lineages have independently evolved functional interactions with the obligatory blood feeders, but, notably, all converge to an analogous biochemical feature of vitamin provisioning.

## Author contributions

All authors listed have made a substantial, direct, and intellectual contribution to the work and approved it for publication.
